# Epigenetic investigation into circulating microRNA 197-3p in sera from patients affected by malignant pleural mesothelioma and workers ex-exposed to asbestos

**DOI:** 10.1038/s41598-023-33116-z

**Published:** 2023-04-20

**Authors:** Giulia Di Mauro, Francesca Frontini, Elena Torreggiani, Maria Rosa Iaquinta, Andrea Caselli, Chiara Mazziotta, Valentina Esposito, Elisa Mazzoni, Roberta Libener, Federica Grosso, Antonio Maconi, Fernanda Martini, Ilaria Bononi, Mauro Tognon

**Affiliations:** 1 Department of Medical Sciences, Laboratories of Cell Biology and Molecular Genetics, School of Medicine, University of Ferrara, 64B, Fossato di Mortara Street, 44121 Ferrara, Italy; 2 Department of Chemical, Pharmaceutical and Agricultural Sciences-DOCPAS, University of Ferrara, 44121 Ferrara, Italy; 3 Research Training and Innovation Infrastructure - Department of Integrated Research and Innovation Activities (DAIRI), AO SS. Antonio e Biagio e Cesare Arrigo, 15121 Alessandria, Italy; 4 Mesothelioma Unit, AO SS. Antonio e Biagio e Cesare Arrigo, 15121 Alessandria, Italy; 5 Department of Translational Medicine and for Romagna, University of Ferrara, 70, Fossato di Mortara Street, 44121 Ferrara, Italy

**Keywords:** Cancer, Genetics, Biomarkers, Molecular medicine, Oncology

## Abstract

The epigenetic role of microRNAs is established at both physiological and pathological levels. Dysregulated miRNAs and their targets appear to be a promising approach for innovative anticancer therapies. In our previous study, circulating miR-197-3p tested dysregulated in workers ex-exposed to asbestos (WEA). Herein, an epigenetic investigation on this circulating miRNA was carried out in sera from malignant pleural mesothelioma (MPM) patients. MiR-197-3p was quantified in MPM (n = 75) sera and comparatively analyzed to WEA (n = 75) and healthy subject (n = 75) sera, using ddPCR and RT-qPCR techniques. Clinicopathological characteristics, occupational, non-occupational information and overall survival (OS) were evaluated in correlation studies. MiR-197-3p levels, analyzed by ddPCR, were significantly higher in MPM than in WEA cohort, with a mean copies/µl of 981.7 and 525.01, respectively. Consistently, RT-qPCR showed higher miR-197-3p levels in sera from MPM with a mean copies/µl of 603.7, compared to WEA with 336.1 copies/µl. OS data were significantly associated with histologic subtype and pleurectomy. Circulating miR-197-3p is proposed as a new potential biomarker for an early diagnosis of the MPM onset. Indeed, miR-197-3p epigenetic investigations along with chest X-ray, computed tomography scan and spirometry could provide relevant information useful to reach an early and effective diagnosis for MPM.

## Introduction

The malignant pleural mesothelioma (MPM) is an aggressive malignancy of the pleural surface/layer. MPM is characterized by poor prognosis, with median survival ranging from 8 to 14 months from diagnosis^1^. The vast majority of MPM cases (> 80%) are linked to asbestos fiber exposure, which mainly occurs in the workplace^2^. However, MPM carcinogenesis is influenced by other factors, too. In vitro and in vivo studies have suggested that other types of mineral fibers, such as erionite, fluoro-edenite, balangeroite and carbon nanotubes can induce MPM^3^. In addition, therapeutic radiation for other malignancies is a well-established cause of MPM, with relative risks as high as 30%^3^. Oncogenic viruses, such as the small DNA tumor simian virus 40 (SV40), have been associated with MPM, suggesting a synergistic action between asbestos fibers and SV40^4^. Moreover, genetic predisposition, involving BRCA-associated protein 1 (BAP1) expression, CDKN2A and neurofibromatosis type 2 (NF2) tumor suppressor gene inactivation, seem to play a strong role as etiological factors that, alone or alongside asbestos, may contribute to the MPM onset and progression^5^.

At present, there are no effective treatments for MPM. Indeed, therapeutic strategies, such as immune checkpoint inhibitors and anti-angiogenic therapies, that showed impressive clinical responses in other solid malignancies, have little impact on MPM^6^. Moreover, MPM is extremely resistant to current chemotherapeutic agents, radiotherapy and some immunotherapy options^7^. Published data estimate an average of 14,200 new cases of MPM worldwide each year^8^. Moreover, global MPM prevalence has increased over the past decade, while its incidence is expected to grow further to an estimated peak in 2025–2030^9^. Although asbestos has been banned in many countries, it should be recalled that in some areas, such as Brazil, India and Russia, its use remains unregulated^2^.

It has been reported that asbestos fibers trapped between the pleural layers and the wall of the chest cavity induce oxidative stress and chronic inflammation, thus promoting a potentially carcinogenesis process^10^. Workers, who were previously exposed to asbestos (WEA), can develop MPM even decades after their exposure to this natural oncogenic mineral^11^. In the early phase of the disease, clinical signs are absent. For this reason, MPM is diagnosed in its advanced stages^12^.

MPM, like many other solid tumors, is a heterogeneous cancer. Histologically, MPM can be classified in three main subtypes, depending on cellular morphology: (i) epithelioid MPM subtype, which represents the most frequent form (50–70% of cases), shows polygonal, oval, or cuboidal cells; (ii) sarcomatoid MPM subtype (10–20%), with the worst prognosis, is characterized by spindle cell morphology; (iii) biphasic MPM subtype (30%), presents a mixture of the two previous morphologies, in different proportions^13^. Histological classification only partially indicates tumor heterogeneity observed at both molecular and clinical levels^14^.

In recent years, alongside histology, different serum proteins have been proposed as potential diagnostic and prognostic MPM biomarkers, such as soluble mesothelin, osteopontin, fibulin-3 and High Mobility Group Box Protein-1 (HMGB1)^15^. Moreover, different investigations have been carried out over the past decade to identify new and less invasive early diagnostic and prognostic markers of MPM, such as circulating microRNAs^16^. Indeed, the use of serum or plasma samples would reduce the invasiveness of current diagnostic techniques^17^. To date, several circulating microRNAs have been proposed as biomarkers for the MPM diagnosis and prognosis. However, at present, there is no consensus on the selection of the best biomarkers. Some of the proposed circulating microRNAs include miR-16, miR-126^18^, miR-103a-3p, miR-30e-3p^19^, miR-29c-3p^20^, miR-486-5p^21^. All identified MPM biomarkers have poor sensitivity and specificity and the majority of them still need to be validated. Moreover, no single biomarker has been proven to work more efficiently than conventional prognostic models^22^. Indeed, studies on microRNAs as biomarkers may show some limitations, including lack of standardization of laboratory techniques, limited sample size, and inconsistency of groups used as controls, such as healthy unexposed subjects, asbestos-exposed subjects with benign diseases, or other cancer patients^16,23^. Therefore, specific, sensitive biomarkers, providing early, effective diagnosis in high-risk subjects need to be identified. To this end, MPM epigenetic analysis has gained increasing attention overtime.

It is well established that gene expression regulation occurs through different mechanisms, including the epigenetic control played by microRNAs. MicroRNAs (miRNAs or miR) are highly conserved small non-coding RNAs, which may cause messenger RNA (mRNA) degradation and/or translational inhibition by binding directly to the 3' untranslated regions (3' UTR) of target mRNAs^24^. Several miRNAs have been implicated in various cellular processes, including cell proliferation, differentiation, stem cell renewal, stress responses, apoptosis and metabolism^25^. Moreover, numerous studies indicate a close association between miRNAs aberrant expression and cancer of different histotypes^26^. The expression analysis of many microRNAs has been investigated in different tumors, including MPM, as these small molecules can be detected in both tissues and biological fluids^27^. Among dysregulated miRNAs, miR-197-3p has been reported to be involved in bladder cancer^28^, thyroid cancer^29^, hepatocellular carcinoma^30^, colorectal cancer^31^ and lung cancer^32^. Additionally, we have recently identified that expression of circulating miR-197-3p was significantly down-regulated in sera from workers who had previously been exposed to asbestos (WEA) compared to sera from healthy subjects (HS), suggesting that miR-197-3p could be proposed as a new potential biomarker for asbestos exposure^33^.

Herein, miR-197-3p expression was assessed in a significantly large-sized sample of sera (N = 225) from MPM (N = 75), WEA (N = 75), and HS (N = 75), using two different PCR approaches, i.e., the innovative, highly analytical, droplet digital PCR (ddPCR) and the consolidated quantitative Real-Time PCR (RTqPCR) The aim of this study was to evaluate miR-197-3p as potential biomarker for a MPM early diagnosis.

## Results

### MicroRNA-197-3p expression level

In our investigation, miR-197-3p detection and its quantification were carried out using ddPCR and RT-qPCR techniques. DdPCR was employed in our investigation because is more sensitive and specific, compared to RT-qPCR^34^.

**Figure 1:**
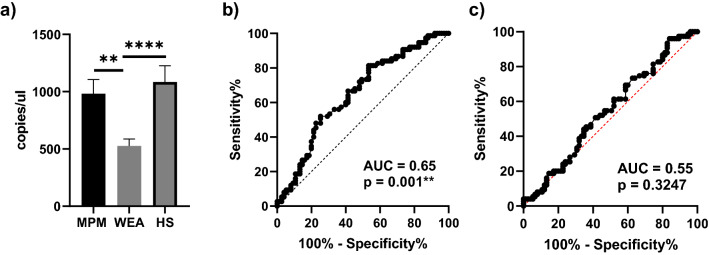
MiR-197-3p analysis by ddPCR. (**A**) WEA and HS cohorts: the amount of miR-197-3p is represented as copies/µl of analyzed cDNA (ANOVA ****P < 0.0001; Tukey’s test: MPM vs WEA **P = 0.0036; WEA vs HS ****P = 0.0001); (**B**) ROC curve for the comparative analysis of miR-197-3p levels in MPM vs. WEA; (**C**) ROC curve for the comparative analysis of miR-197-3p levels in MPM vs. HS.

In the first step of our research, ddPCR analyses detected miR-197-3p in all sera (n = 225). A mean of 981.7 ± 123.7 (mean ± SEM) copies/µl of miR-197-3p was detected in MPM sera, whereas a mean of 525.01 ± 62.13 (mean ± SEM) and 1,085 ± 141.3 (mean ± SEM) copies/µl of miR-197-3p was detected in WEA and HS, respectively (Fig. 1A).

Moreover, multiple comparison indicated a significantly different miR-197-3p expression in MPM cohort compared to WEA (Tukey’s test, P = 0.0036**) and between WEA and HS cohorts (Tukey’s test, P = 0.0001****); while no difference emerged between MPM and HS cohorts (Tukey’s test P = ns). Specifically, miR-197-3p was up-regulated 1.9 times in MPM vs. WEA (Fig. 1). The ROC analysis performed on ddPCR results, revealed that the AUC value for miR-197-3p in the comparison between MPM vs. WEA was 0.65 (P = 0.001**; 95% confidence interval 0.563 to 0.739) (Fig. 1B) while between MPM and HS was 0.55 (P = ns, 95% confidence interval 0.454 to 0.640) (Fig. 1C). The comparison between groups gave highly significant differences (ANOVA P < 0.0001****).

**Figure 2:**
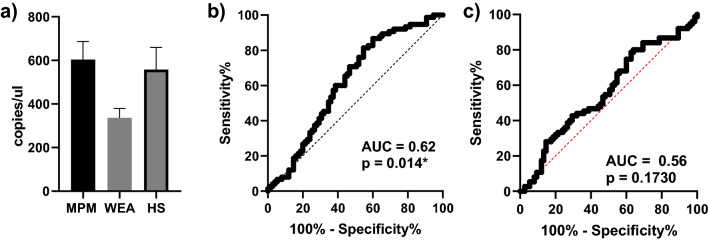
MiR-197-3p analysis by RT-qPCR. (**A**) WEA and HS cohorts: the amount of miR-197-3p is represented as copies/µl of analyzed cDNA (ANOVA *P* = ns; Tukey's test: MPM vs WEA *P* = ns; MPM vs HS *P* = ns; WEA vs HS *P* = ns). (**B**) ROC curve for the comparative analysis of miR-197-3p levels in MPM vs. WEA; (**C**) ROC curve for the comparative analysis of miR-197-3p levels in MPM vs. HS.

MiR-197-3p expression analyzed by RT-qPCR confirmed the differences among groups (MPM > WEA; HS > WEA). However, comparative RT-qPCR analyses of miR-197-3p level in sera appeared not significantly dysregulated in the three cohorts under investigation (ANOVA P = ns) nor in multiple comparisons between individual groups (Tukey's test: MPM vs WEA P = ns; MPM vs HS P = ns; WEA vs HS P = ns). Specifically, a mean of 603.7 ± 82.51 (mean ± SEM) copies/µl of miR-197-3p was detected in MPM sera, whereas a mean of 336.1 ± 43.39 (mean ± SEM) and 557.8 ± 101.1 (mean ± SEM) copies/µl of miR-197-3p was detected in WEA and HS respectively (Fig. 2A). Hence, miR-197-3p was up-regulated 1.8 times in MPM vs. WEA (Fig. 2).

Receiver Operating Characteristic (ROC) analysis was then used to quantify the accuracy of RT-qPCR results in discriminating data in significant comparisons. The ROC AUC for the comparative analysis of miR-197-3p levels in MPM vs. WEA was 0.62 (P = 0.014*; 95% confidence interval 0.525 to 0.708) (Fig. 2B), indicating that miR-197-3p levels significantly discriminate MPM from WEA, while the comparison between MPM and HS was not significant (AUC = 0.56, P = ns, 95% confidence interval 0.472 to 0.657) (Fig. 2C).

As shown in Supplementary Figure S1, linear regression analysis indicated a significant correlation between ddPCR and RT-qPCR values within both study groups. The R-square was 0.879 for miR-197-3p (P < 0.0001****).

**Figure 3:**
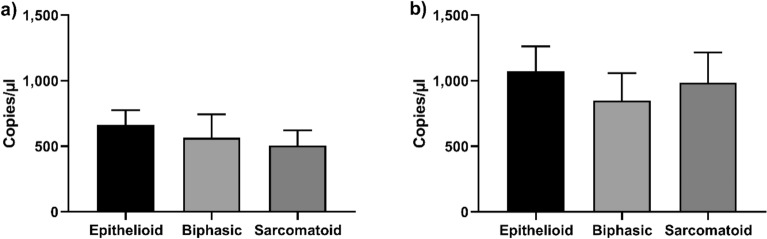
MiR-197-3p levels in MPM histotypes. MiR-197-3p expression detected by RT-qPCR (**A**) and by ddPCR (**B**). The amount of miR-197-3p is represented as copies/µl of analyzed cDNA.

MiR-197-3p expression, assessed by both PCR techniques, did not show significant dysregulation among sera from the three different MPM subtypes, such as epithelioid, sarcomatoid and biphasic, (Fig. 3).

### Clinicopathological data and statistics

In order to investigate putative correlation between MPM clinicopathological data and expression level of miR-197-3p, overall survival (OS) outcome at 18 months was recorded in all 75 patients diagnosed with MPM.

**Figure 4:**
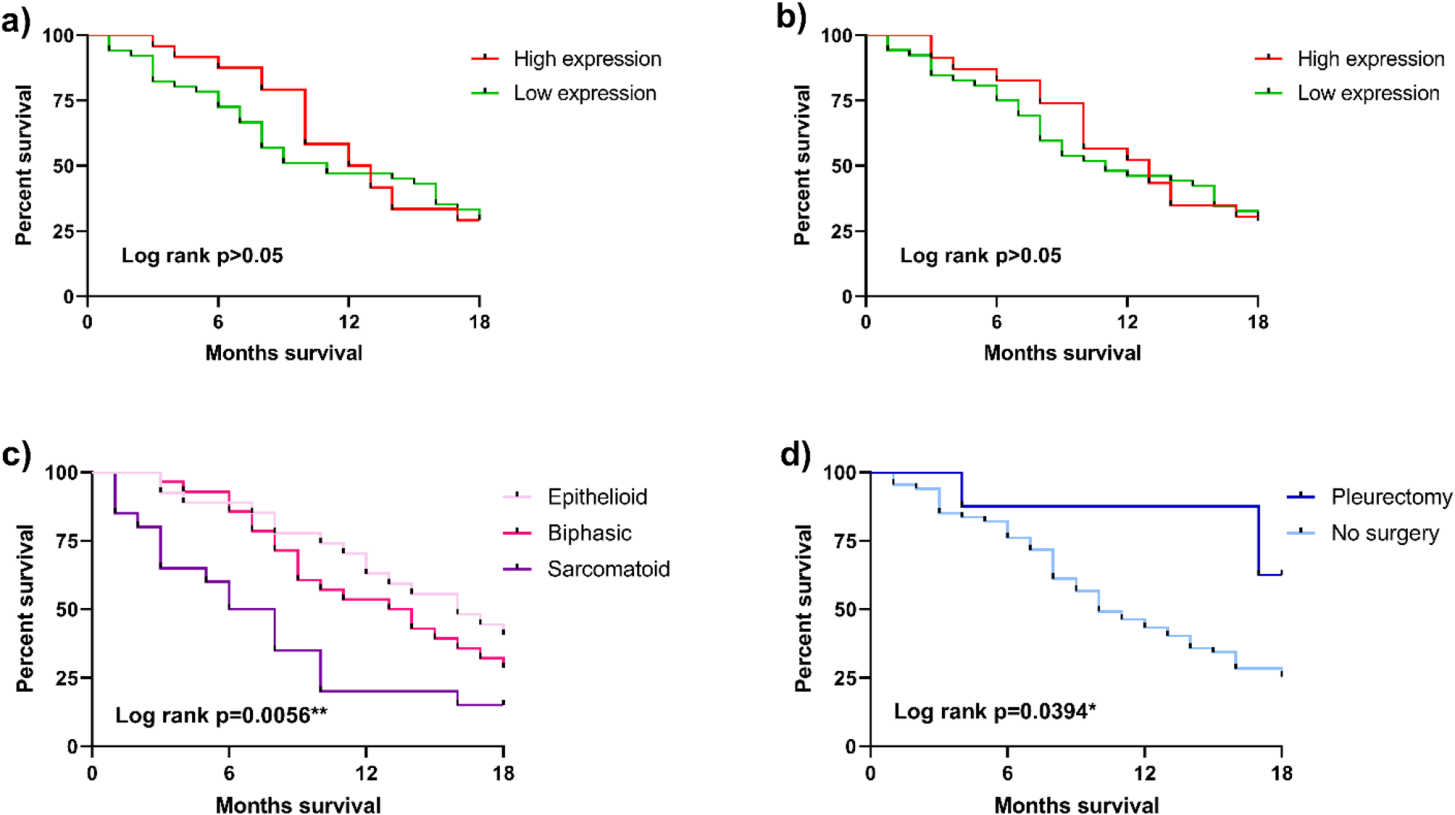
Overall survival (OS) outcome analysis at 18 months in MPM cohort. (**A**) Kaplan–Meier (KM) curves for OS in MPM patients related to miR-197-3p expression revealed by RT-qPCR (log-rank *P* > 0.05) and (**B**) ddPCR (log-rank *P* > 0.05). (**C**) Kaplan–Meier (KM) curves for OS in MPM patients related to histological subtype (log-rank **P = 0.0056). (**D**) Kaplan–Meier (KM) curves for OS in MPM patients underwent to pleurectomy (log-rank *P = 0.0394).

First, the Kaplan–Meier methodology was used to determine the impact of miR-197-3p expression on OS. As reported in Fig. 4A-B, results indicate that OS was not influenced by miR-197-3p expression (P > 0.05). Then, the impact of histological subtype on OS was evaluated. As shown in Fig. 4C, histological subtype (log-rank P = 0.0056**) significantly influenced OS: specifically, OS was longest in patients with epithelioid MPM (40.7%), intermediate in biphasic cases (28.6%), while the shortest OS was revealed in the sarcomatoid MPM subtype (15%).

Subsequently, Kaplan–Meier methodology was employed to determine the impact of pleurectomy on OS in all patients. OS was significantly influenced by pleurectomy (log-rank P = 0.0394*). OS was longest in patients who underwent pleurectomy (62.5%) compared to unoperated patients (25.4%) (Fig. 4D). Finally, the same methodology reported no correlation between OS and asbestos exposure (P > 0.05) or smoking status (P > 0.05) (Supplementary Figure S2).

Multiple linear regression analysis of miR-197-3p expression, related to independent variables, i.e. MPM histological subtypes, asbestos exposure, tobacco smoking status and surgical interventions (Table 1), gave no correlation (data not shown). Moreover, Spearman correlation coefficients were calculated between miRNA quantity and the independent variables evaluated, i.e. clinicopathological characteristics, evaluated. Specifically, in the MPM cohort no correlation was found between miR-197-3p expression and other concurrent pathologies (data not shown).

**Table 1:** Clinicopathological characteristics of MPM cohort.

MPM Cohort–Clinicopathological characteristics		Number of samples (%)
MPM histological subtypes	Epithelioid	27 (36.0)
	Sarcomatoid	20 (26.7)
	Biphasic	28 (37.3)
Asbestos exposure	Professional, documented	17 (22.7)
	Professional, possible	5 (6.7)
	Domestic/Environmental	14 (18.7)
	No exposure	2 (2.7)
	N/A	37 (49.3)
Tobacco smoking status	Smoker	8 (10.6)
	Ex-smoker	13 (17.3)
	Non-smoker	14 (18.7)
	N/A	40 (53.3)
Surgical interventions	Pleurectomy	8 (10.7)
	No intervention	67 (89.3)

Clinicopathological characteristics, occupational e non-occupational information, collected for MPM cohort. It was not possible to obtain all information for all subjects (N/A: data not available).

## Discussion

Many investigations indicate that miRNAs can be considered promising biomarkers for MPM, as well as for other cancers. Indeed, they are detectable in cells, tissues and body fluids of subjects/patients exposed to toxic agents, affected by inflammatory disorders and infectious diseases^27^. Identifying such sensitive and specific MPM biomarkers, alongside markers of asbestos exposure in workers who had previously been exposed to asbestos fibers (WEA), appears of paramount importance for this fatal cancer^9,35^.

To this aim, several studies investigated the diagnostic value of tissue, pleural effusion, and circulating microRNA for MPM^27^. By using microRNA array or public databases, some microRNAs with potential diagnostic significance have been identified. Specifically, mir-126^36,37^, miR-103a-3p^38,39^ and mir-2053^40^ represent the most promising candidates for a MPM diagnosis. However, their diagnostic accuracy, assessed with the ROC curve, is still moderate.

In this study, circulating miR-197-3p was investigated as a potential MPM biomarker, in a significantly large-sized serum sample made up of two cohorts, MPM and WEA. MiR-197-3p, whose gene maps in chromosome 1p13.3, has been reported as being involved in key cellular processes, such as cell proliferation, apoptosis, differentiation, metastasis and drug resistance^41^. Significant miR-197-3p dysregulation has been detected in sera and cells in different human tumors^42–44^. In addition, miR-197-3p has been demonstrated to function as a pro- or anti-oncogenic gene regulator depending on its target gene and target cell lineage^45,46^.

Several studies have reported miR-197-3p as an activated oncogene. In this instance, a different series of target genes have been identified, including p120-catanin, FUS1, tumor suppressor NLK, homeodomain-interacting protein kinase 3 (HIPK3), lysine 63 deubiquitinase (CYLD) gene, EHD2, NOXA and BMF, PD-L1 and Wnt/β-catenin^32,41,47–51^. Conversely, other studies have considered miR-197-3p as a tumor suppressor gene product. Its main target genes are VDAC1, ELK1, FBXW7, KLF10, ZIK1, TYMS, SERPINA3 and TSPAN3, hTERT, E2F6 and RAN (RAS-related nuclear protein)^30,52–58^.

Herein, miR-197-3p was found to be significantly up-regulated in sera from MPM vs. WEA. Interestingly, the two PCR techniques used in this study, ddPCR and RT-qPCR, reported almost overlapping results. Nevertheless, our data showed, as expected, higher sensitivity and specificity for ddPCR than RT-qPCR. These results are consistent with other studies outlining ddPCR as an innovative technique, with lower sensitivity to PCR inhibitors, greater analytical power and reliability in quantifying rare targets from very limited clinical samples^59^.

It is worth noting that ddPCR is an improvement on conventional PCR technique, as it allows for more sensitive, accurate quantification of target nucleic acids^60^. DdPCR overcomes problems of potential discrepancies in PCR analyses: (i) ddPCR performs absolute quantification thanks to the principles of sample partitioning and Poisson statistics, thus overcoming normalization and calibrator issues; (ii) it is relatively insensitive to potential PCR inhibitors; (iii) it is characterized by more precision and sensitivity in detecting low target copies; (iv) it directly provides the result of the analysis expressed as the number of copies of target per microliter of reaction^34^.

The up-regulation of circulating miR-197-3p observed in MPM suggests that miR-197-3p may act as an activated oncogene. On the other hand, a recent study showed the down-regulation of miR-197-3p in hepatocellular carcinoma (HCC) tissues associated with tumor aggressiveness, indicating that this microRNA may be a predictor of prognosis in patients with HCC^30^. Moreover, it has been reported that miR-197-3p plays an inhibitory role in numerous human cancers, such as colorectal cancer, oral squamous cell carcinoma, lung cancer and prostate cancer^31,32^.

In the present work, despite the similarity of miR-197-3p expression levels in MPM and HS sera, this circulating miR seems to be well suited as MPM marker. Indeed, it is unlikely that a putative increase of the miR-197-3p level over time, which can be revealed in an asbestos ex-exposed subject, indicates a "return to normal." As reiterated in the literature, asbestos fibers are never eliminated from the body, once inhaled^61^. An increase of miR-197-3p level in an asbestos-exposed subject could indicate the MPM onset at its early stage. Therefore, miR-197-3p increased level may allow the subject to be monitored more closely than other WEA, where this miR remains stable, to identify the occurrence of the MPM onset. Indeed, ROC curve analysis demonstrates AUCs (0.62/0.65) at the limit of acceptability for a marker, compared with what is reported in the literature (AUC > 0.70–0.80).

In a previous study, we reported that the expression of circulating miR-197-3p was significantly down-regulated in sera from workers who had previously been exposed to asbestos (WEA) compared to HS^33^ and its expression level was inversely correlated with cumulative exposure to asbestos, indicating that miR-197-3p can be considered a new biomarker for exposure to this tumorigenic mineral. Interestingly, comparing the data obtained from MPM reported in this study with those from WEA described in the aforementioned work, the expression of circulating miR-197-3p was higher in the MPM than in the WEA cohort, analyzed by both ddPCR and RT-qPCR, with statistical significance in ddPCR (Tukey’s test, P = 0.0036**). The discrepancy between the two techniques is due to the RT-qPCR, which is less accurate than ddPCR.

It has been established that each microRNA, in addition to regulating a variable number of targets, is also regulated by several non-coding RNAs in a larger network of RNA-RNA interactions^62^. Indeed, miR-197-3p has been shown to have many upstream non-coding RNAs that regulate its expression levels. Some non-coding RNAs act by decreasing miR-197-3p levels through, for example, the sponging mechanism^63^. Others affect miR-197-3p expression by increasing its levels^51,64^. In both cases, variuos studies have observed the involvement of miR-197-3p regulations with drug resistance mechanisms^52,64^.

Therefore, it is interesting for the interpretation of our data to consider this intriguing aspect of miR-197-3p. Overall, MPM is difficult to diagnose as well as to treat. In recent years, strategies for the treatment of MPM have been evolving from chemotherapeutic agents, such as pemetrexed and cisplatin, to monoclonal antibodies or immune checkpoint inhibitors (ICI)^65^.

The significant increase in miR-197-3p levels found in the MPM cohort patients compared to WEA is at present under investigation, including studies related to some mechanism linked to drug resistance. Functional analyses may elucidate the role of miR-197-3p in the MPM onset.

In this study, we also confirm the importance of MPM histological subtypes for patient survival^66^, as the strongest and most consistent predictor of MPM survival. Similarly, we corroborate a significant correlation between patient survival and pleurectomy, as reported in other works^67^. However, our data indicate that miR-197-3p is not correlated with patient survival.

Overall, circulating miR-197-3p was found to be expressed at higher levels in MPM vs. WEA sera. Our results provide a new perspective on epigenetic alterations in the MPM, highlighting a possible role for miR-197-3p as a biomarker of this tumor, understood as a deviation from the WEA status. The interpretation of epigenetic alterations in MPM through miRNAs may contribute to the development of new diagnostic and therapeutic strategies. Further investigations are needed to elucidate its role in patients with MPM, including validation/identification of its target genes and its involvement in asbestos-induced chronic inflammation. The challenge we face, is to understand the role of miR-197-3p and molecules interacting with it in the MPM onset.

It is worth noting that there is still a worrying lack of reliable diagnostic markers for an early MPM onset, prevention and screening, as well as validated targets for developing new therapeutic treatments. MiR-197-3p may represent a new marker to be analyzed both in WEA and MPM sera. Liquid biopsy can be obtained with low invasive methods and this miRNA appears to be a quantifiable, specific, sensitive biomarker.

In conclusion, circulating miR-197-3p could be added as minimally invasive screening test to follow-up WEA, that are individuals at high-risk to develop MPM.

## Materials and methods

### Sera

Serum samples (N = 225) were from malignant pleural mesothelioma affected patients (MPM, n = 75), workers ex-exposed to asbestos (WEA, n = 75) and healthy subjects (HS, N = 75). MPM sera were obtained from the Mesothelioma BioBank, Pathology Unit, City Hospital of Alessandria, Italy, whereas sera from WEA were supplied by the Occupational Medicine Unit, University of Ferrara, Ferrara, Italy, as previously described^33^. HS sera were collected at the Clinical Laboratory Analysis of the University Hospital of Ferrara, Italy. HS sera were taken from discarded laboratory samples before incineration, after routine analyses. HS were subjects in good health at the time of blood sampling, as reported in the hospital records^33^.

Sera were anonymously collected, aliquoted and stored at − 80 °C until the time of analysis. All peripheral blood samples (HS, MPM and WEA) were collected employing the same method, i.e. in tubes with a serum gel separator. Samples were allowed to clot at room temperature for 30 min, then, they were centrifuged at 1,500 g for 10 min at room temperature, within 2–4 h of blood draw to ensure miRNA stability and a good extraction output^68^. After centrifugation, the supernatant was aliquoted into 1.5-mL sterile RNase-free tubes and immediately frozen at − 80 °C until the day of miRNA extraction. For comparative analyses, sera of MPM, WEA and HS were coded with indications of age, gender and pathology, if any. The three cohorts were made up of subjects/patients with similar ages (MPM 69.3 ± 0.85; WEA 66.3 ± 0.78 and HS 69.8 ± 0.92, years ± SD). All study participants signed their written informed consent at the time of the hospital admission. The study was approved by the County Ethical Committee, Ferrara, Italy (ID numbers 151078, 160,986).

Clinicopathological characteristics, occupational and non-occupational information, were collected from WEA^33^ and MPM cohorts (Table 1 and 2). Specifically, for the WEA cohort, data related to asbestos exposure and tobacco smocking status were from our previous study^33^. For the MPM cohort, in addition to asbestos exposure and tobacco smoking status, collected information concern MPM histological subtypes, surgical interventions, pharmacological therapies and concurrent diseases, not related to asbestos.

**Table 2:** Survival data at 18 months for MPM cohort.

MPM Cohort	Histological Subtype	Mean of months + SEM
Survival	Epithelioid	13.5 ± 0.6
	Sarcomatoid	7.9 ± 0.7
	Biphasic	12.4 ± 0.6
	TOT	11.5 ± 0.6

### Ethics approval and consent to participate

The study was conducted according to the guidelines of the Declaration of Helsinki (as revised in 2013). The Ethics Committee of Ferrara approved the study (ID numbers 151078, 160,986) and all study participants signed their written informed consent.

### RNA extraction and miRNA reverse-transcription

Total RNA, including miRNAs, was extracted from 200 μl of serum from each patient/subject involved in the study. The miRNeasy Mini Kit (QIAGEN, RRID:SCR_008539, Milan, Italy) was used following the manufacturer’s instructions, with the following minor modifications. The final amount of miRNA, extracted from serum, may be influenced either miRNA extraction efficiency or RT-qPCR robustness. These factors can be verified by synthetic non-human miRNAs added to the serum sample, employed as controls, before the RNA isolation. In order to adjust these parameters, synthetic cel-miR-39 miRNA (2.5 μl of 5 nM) (QIAGEN, RRID:SCR_008539, Milan, Italy) was added to the serum sample (200 ul), soon after adding the Qiazol Reagent (1 ml) to be used as an exogenous extraction control. RNA was eluted in 30 µl RNAse-free water.

MiRNAs were reversely transcribed using the miRCURY LNA RT kit (QIAGEN, RRID:SCR_008539, Milan, Italy) following the manufacturer’s instructions. Specifically, 2 μl of total RNA was reverse transcribed in a final volume of 10 μl by adding 2 μl of 5X reaction buffer and 1 μl of enzyme mix. Synthetic RNA spike-in UniSp6 was used as an exogenous control of the reverse transcription. Reactions were carried out on the Simply Amp Thermal Cycler (Thermo Fisher Scientific, RRID:SCR_008452, Waltham, MA USA), with the following conditions: 42 °C for 60 min, 95 °C for 5 min, and finally a holding step at 4 °C RT products. cDNAs, diluted 1:60, were stored at − 20 °C until the time of qPCR analyses. Cel-miR-39 and Unisp6 assays were performed to monitor RNA extraction and RT reaction efficacy^69^.

### MiRNA quantification: calibrations and controls

A specific miRCURY LNA miRNA PCR Assay (QIAGEN, RRID:SCR_008539, Cod. 339,306-YP00204380) was used to analyze hsa-miR-197-3p concentrations by RT-qPCR and ddPCR. Standard curves were generated for hsa-miR-197-3p using Specific synthetic Locked Nucleic Acid (LNA) oligonucleotides with the following sequence: synthetic hsa-miR-197-3p 5’-rUrUrCrArCrCrArCrCrUrUrCrUrCrCrArCrCrCrArGrC-3’ (QIAGEN, RRID:SCR_008539, Milan, Italy).

Stock solutions (100 μM) of synthetic oligonucleotides, in RNase-free/DNase-free water, were prepared following the manufacturer’s instructions. In order to construct the standard curve of synthetic oligonucleotides miR-197-3p, serial dilutions of the stock solution were performed at 1:10 from 10^13^ copies/μl to 10^0^ copies/μl. The obtained miR-197-3p standard curve was used to quantify miRNAs absolutely by RT-qPCR^70^ and one point of the curve was used as a positive control in ddPCR.

### Droplet digital PCR (ddPCR)

To allow for more specific, analytical detection of miR-197-3p copies/μl and in order to validate RT-qPCR results, the QX200ddPCR System (Bio-Rad Laboratories, RRID:SCR_008426, Hercules, CA USA) was used for ddPCR analyses as previously reported^71,72^.

Briefly, ddPCR was performed in a total volume of 22 μl by adding 11 μl of 2X EvaGreen supermix (Bio-Rad Laboratories, RRID:SCR_008426, Hercules, CA USA), 10 μl of diluted cDNA template (1:60), and 0.5 μl of the specific miRCURY LNA miRNA PCR Assay. After droplet generation inside the Automated Droplet Generator (Bio-Rad Laboratories, RRID:SCR_008426, Hercules, CA USA), the 96 well-plate was heat-sealed with foil and placed in the SimpliAmp Thermal Cycler (Thermo Fisher Scientific, RRID:SCR_008452, Waltham, MA USA). PCR conditions were: 95 °C for 5 min, then 40 cycles at 95 °C for 30 s and 56 °C for 1 min (ramping rate of 2 °C/sec), and three final steps at 4 °C for 5 min, 90 °C for 5 min and a 4 °C indefinite hold to enhance dye stabilization. Each PCR experiment included a mock sample containing H_2_O as a negative control and the synthetic LNA oligonucleotide used as a positive control. Finally, the QX200 Droplet Reader (Bio-Rad Laboratories, RRID:SCR_008426, Hercules, CA USA), and Quanta Soft software (Bio-Rad Laboratories, RRID:SCR_008426, Hercules, CA USA), were used for data analyses.

### Real time quantitative PCR (RT-qPCR)

RT-qPCR reactions were performed using the miRCURY LNA SYBR Green PCR Kit (QIAGEN, RRID:SCR_008539, Milan, Italy) following the manufacturer’s instructions. Briefly, 3 μl of 1:60 diluted cDNA template was used alongside: 0.5 μl of specific miRCURY LNA miRNA PCR Assay (QIAGEN, RRID:SCR_008539, Milan, Italy), and 5 μl of miRCURY LNA SYBR Green (QIAGEN, RRID:SCR_008539, Milan, Italy), in a total reaction volume of 10 μl. Samples were run using the CFX96 Touch (Bio-Rad Laboratories, RRID:SCR_008426, Segrate, Italy) in triplicate with a mock sample containing H_2_O, which was used as negative control, and a positive control consisting of a standard curve generated using specific synthetic LNA oligonucleotides as reported previously.

The thermal cycler conditions were as follows: denaturation at 95 °C 2 min, followed by 40 cycles at 95 °C 10 s and 56 °C 1 min. A final melting curve step, from 56 °C to 95 °C (ramping rate of 0.5 °C/sec), was carried out to verify the amplified product. Standard curves were run in parallel with samples for absolute quantification of miR-197-3p.

### Statistical analysis

Statistical analyses were performed using Prism 8.0 statistical software (GraphPad Prism, RRID:SCR_002798) version #8, La Jolla, CA, USA). Data distribution was verified using the D'Agostino Pearson test or the Shapiro–Wilk test, depending on the number of the cohort examined. As values did not follow a Gaussian distribution, we performed a log-transformation of data in order to carry out statistical analyses. Untransformed data have been shown for ease of interpretation. One-way analysis of variance (ANOVA) and Tukey's multiple comparisons tests were used to assess differences in the mean values of copies/µl of miR-197-3p in all study cohorts. The correlation between RT-qPCR and ddPCR data was tested using the linear regression model. ROC curves were used to evaluate the reliability of circulating miR-197-3p as a malignant pleural mesothelioma biomarker. All parameters, collected for the MPM cohort, were correlated with patients' overall survival (OS) at 18 months using the Kaplan–Meier model to assess the relationship between miR-197-3p and OS. High and low levels of miR-197-3p have been defined using the mean value of miR-197-3p expression to calculate the cut-off point. This is one of the multiple methods available to determine the cutoff point in overall survival analysis^73^. The Log-Rank Test was used to evaluate group differences. *P* values less than 0.05 were considered statistically significant for all analyses. Therefore, Spearman correlation coefficient was used for correlation analysis between miR-197-3p expression and independent variables, i.e. clinicopathological characteristics.

## Supplementary Information

Supplementary Information.

## Data Availability

The data presented in this study are available on request from the corresponding author.

## Abbreviations

MPM: Malignant pleural mesothelioma
WEA: Workers ex-exposed to asbestos fibers
HS: Healthy subjects
miR: O miRNAs, microRNA
RT-qPCR: Real time quantitative PCR
ddPCR: Droplet digital PCR
ROC: Receiver operating characteristic
OS: Overall survival
