# Supplementary material for: Epigenetic investigation into circulating microRNA 197-3p in sera from patients affected by malignant pleural mesothelioma and workers ex-exposed to asbestos
Source: Sci Rep. 2023 Apr 20;13:6501. doi: 10.1038/s41598-023-33116-z (PMC10119131; doi:10.1038/s41598-023-33116-z)
Supplement: Supplementary file 1 — Supplementary Information. [file 41598_2023_33116_MOESM1_ESM.pdf]

Epigenetic investigation into circulating microRNA 197-3p in sera from patients affected by malignant pleural mesothelioma and workers ex-exposed to asbestos

Giulia Di Mauro[1,+], Francesca Frontini[1,+], Elena Torreggiani[1,†], Maria Rosa Iaquinta[1], Andrea Caselli[1], Chiara Mazziotta[1], Valentina Esposito[1], Elisa Mazzoni[2], Roberta Libener[3], Federica Grosso[4], Antonio Maconi[3], Fernanda Martini[1], Ilaria Bononi[5,*], Mauro Tognon[1,*]

Figures S1 and S2 and their captions

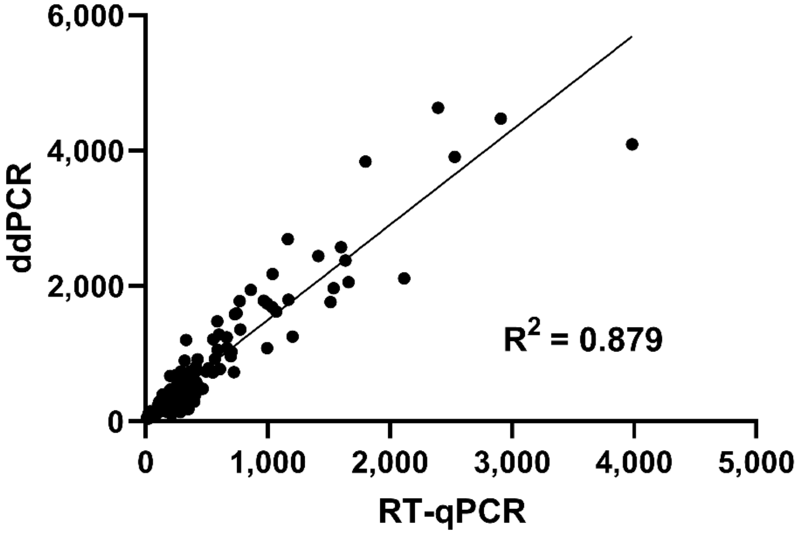

Figure S1. **Correlation between RT-qPCR and ddPCR measurements of miR-197-3p levels in sera.** Linear regression analysis between qPCR and ddPCR values. MiR-197-3p (copies/µl) was measured by both qPCR techniques in 225 samples. R-square = 0.879 (P<0.0001***).

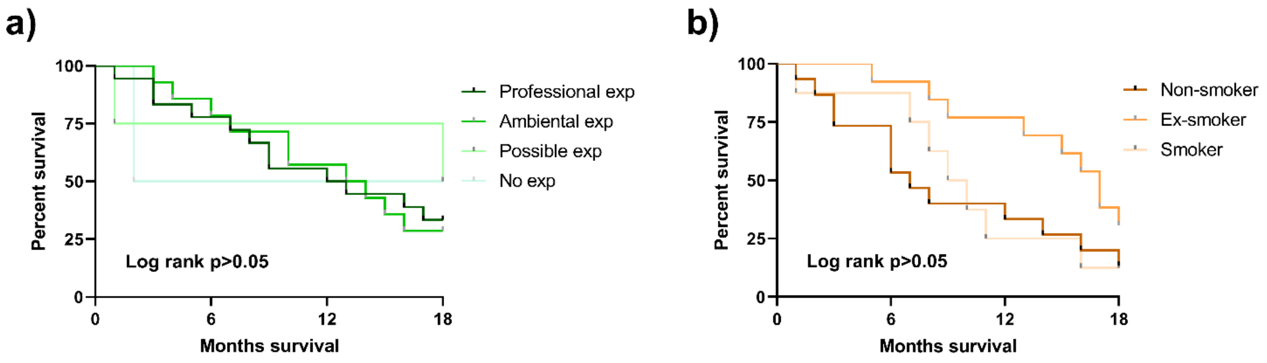

Supplementary Figure S2. Analysis of overall survival (OS) at 18 months related to asbestos exposure and smoking status of MPM cohort. (A) Kaplan-Meier (KM) curves for OS in MPM patients related to asbestos exposure (log-rank p>0.05). (B) Kaplan-Meier (KM) curves for OS in MPM patients in correlation with tobacco smoking status (log-rank p>0.05).
